# On the Means of Purifying the Atmosphere from Contagious Matters, and of Preventing the Progress of Infection

**Published:** 1802-12-01

**Authors:** Guyton Morveau


### *On the Means of purifying the Atmosphere from contagious Matters, and of preventing the Progrefs of Infection; by Cit.* GUYTON-MORVEAU.

ATTEMPTS have repeatedly been made to purify the atmofphere when corrupted by putrid miafmata; but the refearches and experiments hitherto made, and the different expedients generally adopted for that purpofe, have, upon being impartially examined, proved ufelefs, and on the whole, without the intended fuccefs; and the only prerogative they can boaft of, is that of being eftablifhed by the authority of cuftom and inveterate prejudice. It was referved for the fagacity and genius of Cit. Guyton, to difcover the moft efficacious remedy againft the fatal mixture of putrid miafmata with atmofpherical air, and thus to fave the lives of many who would formerly have fallen a facrifice to the penetrating poifon of contagious effluvia.

The vaults of the cathedral in Dijon being entirely filled up with dead bodies, it was ordered that they fhould be emptied, particularly with a view of making them the repofitory for the dead, who, in the hard winter of 1773, could not be buried in the ground, on account of its being fo hardened by froft, as to render digging impoffible. On this being opened, therefore, a ftench immediately arofe, which became fo intolerable, that the church was neceffarily fhut, and the work difcontinued. Recourfe was inftantly had to all the common modes of purifying the atmofphere; but though the ftench of the putrid effluvia was for fome time removed, it appeared again with renewed force, and fpreading itfelf through the neighbouring houfes, traces of its effect on the human conftitution were foon perceived, and infective fevers began to make their appearance. Under thefe embarraffing circumftances, Cit. Guyton was confulted, to propofe an expedient by which the alarming progrefs of this infection could be checked, and the fatal confequences of thefe effluvia powerfully obviated. Cit. Guyton, prompted by an opinion that all putrid miafmata of an offenfive fmell are impregnated with ammonia, which in fact feems to form their vehicle, thought the muriatic acid might be employed with hopes of fuccefs, it being, on account of the great volatility of its fumes, beft calculated for combining itfelf with the ammonia; and that the putrid miafmata, thus difengaged from their vehicle, would be readily precipitated and fall to the bottom.

In confequence of this idea, Cit. Guyton recommended fumigations with muriatic acid, with the view of neutralizing thofe effluvia; and the firft trial was accordingly made with 6lb. of common falt, and 2lb. of concentrated fulphuric acid, which being properly mixed, were put into a glafs bell, placed in a fand bath, and the whole was gradually heated, by which means muriatic fumes were difengaged. The experiment fucceeded beyond the moft fanguine expectations; the beneficial effects of thofe fumigations manifefting themfelves in fo fhort a time, that on the fourth day the church could be opened again for fervice. Another opportunity of trying thefe acid fumigations occurred foon after in the fame place. The atmofphere in the jail of Dijon became fo corrupted, that a fort of malignant jail fever broke out among the prifoners, by which many of them were carried off; but the apartments being fumigated with muriatic acid, only for one day, the atmofphere was fo much purified, that a young furgeon flept the whole night in one of the moft infected prifons without experiencing the leaft harm. Similar fumigations were likewife found of great efficacy in a diftemper of the horned cattle, which in the year 1774 raged in the South of France; and though fumigations with aromatic fubftances, woods, herbs, and refins, were at the fame time employed, the principal effect, however, was obtained from the fumes of the muriatic acid, by which the ftench of the atmofphere was almoft inftantaneoufly removed, and the air rendered pure and falutary.

Induced by thefe facts, the Council of Health, (Confeil de Santé) iffued orders that the fumigations with muriatic acid fhould be ufed in military hofpitals, in fhips, in diftempers of cattle, &c.; but as, unluckily, fome neglect took place in publifhing the proper proceedings, and to bring this new method of purifying the atmofphere into more general notice, it feems not to have fo much engaged the attention of the public in France as it really deferves. Similar fumigations have, fince that time, been tried in other countries, particularly in England, the hiftory of which we find recorded in a work publifhed by by Dr. Carmichael Smith. This gentleman, and Mr. Archibald Menzies, employed the nitrous fumes with the view of preventing contagion, and checking its progrefs; in which attempt they perfectly fucceeded, as malignant infective fevers were fuccefsfully repreffed on board of feveral fhips of war, by repeated fumigations with nitric acid. But in this procefs great care muft be taken, that there is no nitrous acid (acide nitreux) formed, which being inhaled, may occafion moft difagreeable fymptoms; whereas the nitric fumes are imbibed without the leaft inconvenience. Mr. Cruickfhank has alfo employed acid fumigation for the fame purpofe; but he admits that all mineral acids are inferior to the oxydated muriatic gas, which is very eafily difengaged by pouring concentrated fulphuric acid on a mixture of two parts of common falt and one part of manganefe, which muft previoufly be diluted with water. Thick fumes will almoft inftantly arife, which uniting with the putrid miafmata, purify the atmofphere in a fhort fpace of time. Muriatic fumigations have been likewife made in Spain, with the view of checking the progrefs of contagion among men and cattle. Cit. Guyton having thus far propofed the hiftorical facts relative to the ufe of fumigations with mineral acids, proceeds to enumerate a feries of experiments, in order to inveftigate the nature of putrid miafmata, and to afcertain the efficacy of different fubftances, particularly of the acid fumes, in correcting the atmofphere, and in repreffing the fatal action of thofe miafmata on animal bodies. With this view he made feveral experiments on air, in which beef had for fome time been fuffered to rot, by which means it had acquired all the requifite properties of corrupted air. It makes lime-water, a folution of nitrat of filver or of mercury, immediately turbid, retaining, notwithftanding, its putrid fmell, a circumftance which certainly proves, that, though it contains a greater proportion of carbonic acid than the atmofpherical air, its properties are quite independent of this part of its mixture. Pieces of paper which were coloured with different vegetable fubftances and with a folution of copper, when placed in that air for fome time, did not change their colour, but only became a little paler than they were before. Several oxyds of metals, viz. of zinc, manganefe, and of lead, were for fome days brought in contact with this air, without either changing their colour or difengaging any ammonia. From thefe experiments it appears, that putrid air contains no free ammonia, as had been formerly fuppofed by Cit. Guyton. At laft he made fome eudometrical experiments on this air, from which it was found to contain almoft as much oxygen as the atmofpherical air; whence its noxious qualities feem not to originate in a want of oxygen. In order, therefore, to examine the nature of the effluvia which render the air putrid, recourfe ought to be had to a chemical analyfis; but all the expedients which the chemical art offers for that purpofe, are infufficient, and we muft content ourfelves with fuppofing that thefe effluvia are compound fubftances, to which the atmofpherical air ferves as a vehicle; and that confequently they are to be decompofed and deftroyed by proper reagents. With this view Citizen Guyton brought the putrid air in contact with fuch ftrong fmelling fumes as are difengaged on burning benzoin and aromatic plants, and fhook it with folutions of myrrh, benzoe, and balfamus Peruvianus in fpirit of wine; but the putrid fmell could not be removed in this manner. Gunpowder proved, likewife, without effect, which being fet on fire in a balloon filled with putrid air, made it move, but without purifying it. It was then mixed with the anti-peftilential preparation, known under the name of thieves vinegar, *(vinaigre de quatre voleurs)* and with brown vinegar *(vinaigre rouge)*; but the putrid fmell was ftill to be perceived twenty-four hours after. Fumes of acetous acid, however, deprived the air of that fmell in a fhort time. Sulphuric acid had not the leaft influence on the putrid air; but the ftench was confiderably diminifhed by fulphurous acid, without being entirely removed. Nitrous and muriatic fumes foon deftroyed the putrid fmell; but in employing the firft, it was difficult to prevent the formation of nitrous acid. Oxydated muriatic gas, however, was moft efficacious in taking away all the fmell in a very fhort fpace of time, and exhibits, undoubtedly, the fureft means of checking contagion. The muriatic acid deferves to be ranked next, as it requires too much trouble to prepare acetic acid in a fufficiently pure ftate, and as with the nitrous fumes a portion of nitrous acid is almoft always difengaged.

After having thus ftated the experiments, with different fubftances, propofed for the purification of the atmofphere, and the confequences which refult therefrom, Cit. Guyton proceeds to examine the influence which the oxygen feems to exert in the procefs of removing infection; and he endeavours to determine whether all contagious miafmata are equally attacked by the fame agents. Amongft the properties of oxygen which chemiftry has revealed to us, it is obferved, that this matter poffeffes a particular difpofition to enter into a combination with a number of fubftances, chiefly with animal bodies; whence it was natural to conclude, that it might be ufed as a medicament, efpecially as it is capable of producing fuch changes in the animal body, by which any morbific matter is diminifhed and due order reftored to the animal functions. With a medical view, however, it ought to be employed in a ftate of combination, tion, when its action is not restrained; and it is on this account, that certain substances prove in proportion efficacious and medicinal, from the more oxygen they contain, and the more easily they yield it to the animal matter. The different medical agents offer undoubtedly a scale of action beginning from the slightest alterative, and rising to the most violent corrosive. The theory, in which the medicines are considered in this point of view, has been adopted by several celebrated physicians of England, in consequence of which some of them have even comprehended all remedies under two general heads, of suroxygenating and desoxygenating remedies, and they have compared their action with the process of combustion. Although the common phenomena of combustion are not to be observed in the combination of oxygen with several bodies, there exists a great analogy in the results of these two processes, as by means of the oxygen, the bodies to which it is conducted suffer several changes in their respective properties, according to the degree of affinity which they have to the oxygen. The changes which perpetually proceed in the animal matters of the living body are really effected by such a slow combustion, that they may be promoted by bringing a great portion of oxygen, or such substances which are endued with much oxygen, in contact with them. By a similar theory, modern physicians were led to employ, with a medical view, the oxygenated unguent, the nitric acid, oxygenated muriatic acid, oxygenated muriat of pot-ash, and several others, which enable us to cure some diseases that formerly resisted other remedies, and were but with difficulty cured.

When it is therefore ascertained, that oxygen has such a remarkable effect on animal substances, it is easily conceived how the putrid miasmata, which are exhaled from them, can likewise be subject to the action of this agent, which combining with them must necessarily change their properties. Cit. Guyton enlarges here, according to this idea, on the question, how the oxygenated substances can prove preservative against infectious diseases; and from which it seems to result that oxygen, and particularly substances which emit it in form of a gas, appear to act in a double way; first, by having an affinity to the contagious miasmata, which decomposes them; and secondly, by augmenting the vital powers and organic action, which enables them to resist the noxious effect of contagion, and particularly its great power of assimilation, which renders it so very dangerous. It is known, that nothing increases and promotes contagion so much as debility; oxygen gas, therefore, acting as a stimulant, by entering the body through the organs of respiration, and by touching its whole surface, resuscitates the activity and sensibility of organization, and thus checks the progress of infection.

 Another

Another queſtion remains to be determined, whether the ſame remedies are applicable in the different ſpecies of contagion? All infectious diſeaſes are not to be derived from the ſame matter, as the ſymptoms with which they are attended, prove their being of a different nature, and aſſign to each of them a particular character. The moſt general difference by which they are to be diſtinguiſhed, is, that ſome infectious diſeaſes originate from effluvia ſpread in the atmoſphere; whereas others, of a more fixed nature, are only communicated by immediate contact. The firſt claſs of contagious diſeaſes is that which muſt particularly engage our attention, it being more difficult to guard mankind againſt them, and in which the neceſſity of preſervatives is chiefly required. To this claſs belong the hoſpital and gaol fever, fevers from marſhy effluvia, and other malignant fevers ariſing from putrid exhalations.

With reſpect to the ſecond claſs of contagious diſeaſes, which are communicated by immediate contact, it is certain that they originate from a particular morbific matter, which is by no means a ſimple ſubſtance, but compound, and conſequently incapable of reſiſting combuſtion when brought in contact with oxygen, to which, like other productions of animal organization, it has the greateſt affinity. The nature of theſe terrible compoſitions, of which the contagious effluvia conſiſt, has not as yet been penetrated; but as the infectious matters poſſeſs the property of multiplying and reproducing themſelves, they cannot be ſimple bodies. It is however not improbable, that azote is the principal conſtituent of infectious matters, and that their ſpecific difference ariſes from the different nature and proportion of thoſe ſubſtances, which ſerve as a vehicle to it; in other words, their different degree of virulence depends on the different degree of *sur-azotation.* It may be thence ſuggeſted, that thoſe morbific matters do likewiſe ſuffer great changes by oxygen and oxygenated ſubſtances; a concluſion which is proved by the evidence of facts and experience. The variolous poiſon is undoubtedly one of the moſt contagious and of a very ſpecific nature; but from the experiments of Mr. Cruikſhank it appears, that when previouſly mixed with oxygenated muriatic acid, and inoculated, it did not produce the leaſt effect; while another portion of the ſame matter, which was inoculated without being mixed with the acid, communicated the ſmall-pox. It is likewiſe known, that the venereal poiſon is deſtroyed by oxygenated mercurial remedies, without which it would have produced ulcers and other venereal ſymptoms. The hydrophobia, which has hitherto been thought incurable when advanced to a certain degree, may be ſucceſsfully treated with oxygenated ſubſtances, applied on the wound, before the local irritation of the nerves has has produced the rabid or hydrophobous. ſymptoms The exanthematous (pſoric) matter has alſo been found to be deſtroyed by the action of oxygenated ſubſtances.

Although we have not yet received any obſervations of the efficacy of oxygenated ſubſtances againſt the infection of the plague, it is more than probable that it may likewiſe be decompoſed by oxygen, and thus deprived of its malignity. Cit. Guyton recapitulates, in the fourth part of his work, the reſults of his experiments with all the means of purifying the atmoſphere that have been to this preſent time employed, to each of which he aſſigns ſuch a rank as it ſeems to deſerve.

1. Cold and warm water may diminiſh and dilate the fetid ſmell of the infectious matter, but are not capable of decompoſing it, and it makes but a new vehicle of the infectious matter.

2. Lime is very uſeful by decompoſing the animal matters before the putrefaction begins, and by abſorbing the carbonic acid; but air charged with putrid miaſmata does not become freed from them by paſſing through lime water.

3. Reſinous ſubſtances, even thoſe from which a volatile acid may be obtained by diſtillation, have no other effect upon the infectious atmoſphere but that of hiding, and as it were maſking the infectious ſtench, without deſtroying the contagious corpuſcles and purifying the air, in whatever manner they may be employed.

4. Fires, which are frequently kindled in infected places, may occaſion a current of air, by which means the ſtagnating ſubſtances are diſperſed; but they prove, in other reſpects, more noxious than ſalutary, and no putrid particle is decompoſed by them, except thoſe which are within reach of the heat, by which their combuſtion can be effected.

5. Subſtances, thrown on burning coals, pure or aromatic vinegars, nitre, and gunpowder, likewiſe never anſwer their intended purpoſe. This is the ſame caſe with ſulphur, the combuſtion of which being never complete, and producing but a ſlight degree oxydation, occaſions a ſulphurous vapour, which though acting efficaciouſly on the infectious matters within its reach, does not ſpread itſelf to a great diſtance, and would be inſupportable in places that are inhabited. It may however be ſucceſsfully uſed for freeing from infection houſehold wares and utenſils, and even during the night time, incloſed and uncovered places, as the ſmall courts of hoſpitals. To this end, powdered ſulphur is placed on an earthen plate, with a ſmall match in the middle, which is kindled.

6. Common vinegar or acetous acid having little expanſibility, even when expoſed to the action of heat, cannot be advantageouſly employed in fumigations; but it is of the

 greateſt greateſt ſervice, if the infected bodies are plunged into or waſhed with it.

7. The liquid pyroligneous acid has an action analogous to that of vinegar, but in a ſtill leſs degree; it is diſengaged by the combuſtion of ligneous ſubſtances.

8. Pure acetic acid, or radical vinegar, has a very great and rapid action on contagious miaſmata, its penetrating ſmell and exhalation changing not only the ſtate of the ſurrounding atmoſphere, but ſtimulating the vital powers to a degree of energy capable of reſiſting the impreſſion of infection. The only inconvenience in employing fumigations of this acid conſiſts in its being with much difficulty obtained in a pure ſtate without great expenſe, a circumſtance by which it is leſs calculated for being uſed on a large ſcale in hoſpitals, ſhips, &c.

9. It is generally acknowledged that the mineral acids are antiſeptic, reſiſting vegetable as well as animal fermentation, and that they are capable of decompoſing the contagious poiſon; in which property however they materially differ amongſt each other, a circumſtance which makes them more or leſs proper for being employed as preſervatives againſt infection. The ſulphuric acid, for inſtance, is not capable of purifying the atmoſphere on account of its being too fixed. The ſulphurous acid produces likewiſe very little effect, except in form of gas, as is the caſe with the combuſtion of ſulphur. Nitrous acid acts only on the reſpirable part of the air, and its vapours are ſuffocating. Nitric acid, however, efficaciouſly deſtroys the putrid miaſmata, but it extends itſelf inconſiderably and ſoon condenſates again; it likewiſe emits nitrous gas, which is attended with great inconveniency to the perſons who inſpire it. The operation of fumigating with nitric acid muſt therefore be often repeated, if it is intended to anſwer its purpoſe. Of all mineral acids the muriatic acid is the moſt beneficial, particularly on account of its incredible expanſibility, a property which comes every where in contact with the miaſmata, which is very eſſential in this operation. The method of employing it is ſimple and not at all expenſive. On adding to the mixture, which will be hereafter indicated, a ſmall portion of oxyd of manganeſe, the oxygenated muriatic gas will be obtained, which undoubtedly deſerves to be firſt ranked as the ſafeſt and ſureſt anticontagious remedy. A very powerful ſubſtance, which has been already propoſed as a preſervative by Vicq d' Azyr, is the oxygenated muriat of tin, (liquor fumans of Libavius): It emits very irritating vapours, and is conſequently to be alſo recommended as a preſervative, though the ſimple muriatic fumigations are procured with leſs trouble and inconvenience. Cit. Guyton terminates his work with a deſcription of the anticontagious contagious and preservative procefs, which are briefly the following.

The fumigations with muriatic acid are performed in places not inhabited at the time in the following manner. Put in the middle of the place which is to be purified, a chaffing difh, on which a pan half filled with fand, or afhes, is to be laid, and place on this bath a glafs or earthen bowl, containing muriat of foda, (common falt). Having heated the bath, pour on the falt, at once, fulphuric acid; which having done, retire immediately, and let the windows and doors be exactly fhut.

The proportion for a high and fpacious ward of twenty beds is, of common falt 9 ounces 6 drams, and of fulphuric acid 7 ounces 7 drams, which quantity is to be augmented or diminifhed according to the fpace that is to be purified.

The fumigations in places which are inhabited, and near the fick beds muft be managed in a different manner. To this end, the apparatus from which the falutary vapours proceed, is made portable, and the fulphuric acid by degrees poured on the falt, by which means the gafous acid is equally fpread, and can be fo managed as not to caufe any inconvenience to the patients. A fmall chaffing difh with burning coals in it, and a Heffian crucible, will properly anfwer this purpofe; into this a fmall quantity of common falt is put, on which, when it begins to be heated, the fulphuric acid is dropped by degrees. Cold fumigations may be likewife made when there is any danger of the fire, and for the greater convenience of private or family ufe. To this purpofe you may apply a fmall glafs bottle with a glafs ftopple, containing fulphuric acid, a large beer glafs, and common falt. The glafs being placed on the ground, put into it a good table fpoon full of falt, and pour on it, at three or four different times, a fmall quantity of the acid, and at each time a great quantity of vapours will be difengaged.

The fumigations with oxygenated muriatic acid are made in the fame manner, and nothing but a fmall quantity of oxyd of manganefe is added. The moft convenient proportion is the following.

| | oz. | dr. | gr. |
|---|---|---|---|
| Common falt - - - | 3 | 2 | 10 |
| Black oxyd of manganefe | 0 | 5 | 17 |
| Water - - - - - - | 1 | 2 | 33 |
| Sulphuric acid - - - | 1 | 7 | 50 |

The falt and manganefe being mixed by trituration, are put into a glafs bowl, and the water being added, pour to it fulphuric acid. This portion is fufficient for a room with ten beds.

Such

Such are the proceedings for purifying the atmofphere by means of muriatic gaffes, which, befides their great ufe, have the additional advantage of being attended with a very trifling expenfe.

